# Prince of Wales's Hospital Fund for London

**Published:** 1900-12-22

**Authors:** 


### The Institutional Workshop.

### PRINCE OF WALES'S HOSPITAL FUND FOR LONDON.

### MEETING OF THE GENERAL COUNCIL.

A meeting of the General Council of the Prince of Wales's Hospital Fund for London was held at Marlborough House on December 18th (H.R.H. the Prince of Wales in the chair) to receive the report of the Distribution Committee.

The following members were present:—The Chief Rabbi, Viscount Duncannon (Hon. Secretary), Lord Rowton, Lord Rothschild (Treasurer), Lord Reay, the Lord Mayor, the Governor of the Bank of England, the President of the Royal Society (Lord Lister), the President of the Royal College of Surgeons (Sir William MacCormac, Bart.), Right. Hon. C. Stuart Wortley, Q.C., M.P., Sir Trevor Lawrence, Bart., Sir Henry Burdett, K.C.B., Mr. John Aird, M.P., Dr. Collins, Mr. H. C. Smith, Mr. J. Pierpoint Morgan, jun., Mr. Julius Wernher, Mr. F. M. Fry, Mr. J. G. Craggs (Hon. Secretary).

Letters regretting their inability to attend were received from the following:—Lord Farquhar, the Bishop of Southwark, Cardinal Vaughan, Rev. J. Guinness Rogers, Lev. T. Bowman Stephenson, Dr. Church (President of the Royal College of Physicians), Mr. Sydney Buxton, M.P., Mr. William Latham, Q.C., Mr. Albert G. Sandeman.

Lord Rothschild (Treasurer) presented a statement of the financial position of the Fund up to December 14th, which showed that up to that date the Fund had this year received £26,888 in annual subscriptions, £8,523 from donations, undefined contributions, and newspaper collections, £6,038 from investments, £1,000 from a legacy from the late Madame Rosa Ann Deléglise, £1,000 as an annual grant from the trustees of the London Parochial Charities for the Maintenance of Convalescent Hospitals, and other smaller sums, making a total of £43,468 13s. 1d. He had been informed that the Fund would also receive £6,000 from the League of Mercy this year, and he congratulated His Royal Highness upon such a substantial contribution, which showed His Royal Highness's wisdom and foresight in forming the League. (Hear, hear.) It was highly satisfactory to him to be able to report that, notwithstanding the absence of any appeal for eighteen months until a few days ago, the annual subscriptions were so far £1,200 more this year than last. It was natural to expect some falling off in donations, which so far this year are £5,000 less, after including those amounts mentioned in the Executive Committee's report. In addition to the legacy he had mentioned above, there was another of £3,000 from the late Sir Henry P. Turner Barron, which the Fund might expect to be paid in 1901.

His Royal Highness moved, and the Lord Mayor seconded, the adoption of the Treasurer's report, which was agreed to.

H.R.H. The Prince of Wales said: "We have now to receive the reports of the Executive and Distribution Committees. From these reports it will be seen that, in consequence of the unusual needs of the several hospitals, we propose to distribute £50,000 this year. This is more than the net funds received so far this year, but if we are able to procure an additional sum of about £5,000 before the end of the year, this £50,000 can be distributed amongst the hospitals without entrenching at all upon the invested funds. I am pleased to note that the Fund has received £1,000 by way of legacy, and will receive £3,000 more early in the new year."

Mr. Hugh C. Smith presented the report of the Executive Committee, which was as follows:—

The Executive Committee have the honour to inform your Royal Highness that, after very careful consideration of the circumstances of the present year and the need of London hospitals for increased support, they have unanimously decided to recommend to your Royal Highness that the sum of £50,000 should be distributed this year, of which £49,000 to be awarded to hospitals and £1,000 to convalescent homes. This is an increase of £8,000 on the amount distributed last year, and last year was an increase of £9,000 upon its predecessor. It is satisfactory for us to be able to report progressive help to the hospitals from this fund.

The amount distributed this year will, unless some special gifts are received before the 31st of December, somewhat exceed the net contributions received, but for the reasons already mentioned your Committee thought it desirable to recommend £50,000 to be distributed this year. The shortness of funds, it is hoped, will not be much, as we have within the last few days received, among other contributions, the following:—Mr. J. Wernher, £2,000; Mr. A. Beit, £500; Mr. H. L. Bischoffsheim, £500; Mr. Max Michaelis, £500; Mrs. T. J. Williams, £300; Mr. Lionel Phillips, £100; Mr. Carl Meyer, £105; the Earl of Sandwich, £100; the Viscount Duncannon, £52 10s.; Duke of Norfolk, K.G., £50; Lord Lister, £20. The League of Mercy will contribute the sum of £6,000 this year.

Not having made any appeal for 18 months, in consequence of the great claims upon public charity for the War and Famine Funds, your Committee thought it would not be unreasonable to make an appeal in November; but in the short time that has elapsed the result obtained has not realised the hopes of the Committee. It must be borne in mind, however, that a large proportion of the charitable public are already subscribers to this Fund, and have had during the year great claims upon their generosity.

Great care has been taken to keep the expenses as low as possible, and it is anticipated that the expenses this year will be only a little more than those of the preceding year, notwithstanding the growth of the work done in the secretarial offices in connection with the annual visiting of the hospitals.

Eighty-seven hospitals applied for a grant from this Fund, of which eighty-two were deemed to be within the scope of its distribution. The whole of these hospitals were visited during the year. The Visiting Committee was this year increased to twenty-seven members, and consists of the following gentlemen:—Lord Lister, Sir William MacCormac, Bart., P.R.C.S.; Sir Thomas Smith, Bart.; W. S. Church, M.D., P.R.C.P.; John Croft, F.R.C.S.; A. B. Duffin, M.D.; Christopher Heath, F.R.C.S.; N. C. MacNamara, F.R.C.S.; Dr. Pavy, F.R.S.; Dr. E. C. Perry; T. Pickering Pick, F.R.C.S.; H. Power, F.R.C.S.; Alfred Willett, F.R.C.S.; Viscount Duncannon, C.B.; Lord Sandhurst, G.C.I.E.; Sir Trevor Lawrence, Bart.; Sir Henry Burdett, K.C.B.; Thomas Burt, M.P.; Charles Burt, J. G. Craggs, F.C.A.; Frederick M. Fry, W. Doure Hoare, H. J. King, James C. Marshall, J. Pierpoint Morgan, jun., Albert G. Sandeman, Hugh C. Smith.

The reports of this Committee were laid before the Distribution Committee, and were considered by them in recommending grants.

The reports of the Distribution Committee and of the Convalescent Homes Committee, both of which have been approved by the Executive Committee, are appended.

It may be mentioned that the Worshipful Company of Drapers, when forwarding an annual subscription of £1,000 during the pleasure of the Court of Assistants in July last, said:—"The Company learnt with much satisfaction from the last report of the Prince of Wales's Hospital Fund that the Committee have appointed visiting sub-committees, consisting of persons practically acquainted with hospital management, with the object of obtaining information as to the merits and needs of the various institutions. The Company regard this as a most important step towards promoting the well-being of metropolitan medical charities and strengthening their claims on public benevolence." Further progress has been made by the movement for the abolition of the heavy rating of the metropolitan hospitals. Sir Savile Crossley having gone on active service to South Africa, his place during his absence has been taken by Viscount Duncannon. In conclusion the Committee would again desire specially to convey their thanks to the Press and other supporters of the Fund for the assistance which has been given to it this year.

(Signed) Hugh C. Smith.

Lord Lister, in presenting the following report of the Distribution Committee for the year 1900, said that he had never felt so much impressed with the importance and value of the services rendered to the hospitals of London by the Fund as he had been this year:—

"The past year has been one of increasing usefulness of the Fund. The hospitals concerned have again been visited by sub-committees consisting of medical men of wide experience and laymen who take special interest in hospital management. These visitations, while they have admirably answered their immediate object of supplying reliable information regarding the merits and needs of the various institutions, have proved of very high value in stimulating improvements both in construction and in all departments of administration. These improvements have almost invariably been cheerfully undertaken by the hospital authorities.

"The Committee have in several cases recommended that the grants from the Fund should be conditional upon the carrying out of measures which they deem important. The suggestions of the visitors have been received in a most friendly spirit.

"This year special attention has been directed to the pressing needs of South and East London, and the grants recommended have been accordingly substantially increased to various institutions situated within those districts.

"The attention of the Committee has been directed to the objections, hygienic and general, which apply to a requirement enforced by some hospitals whereby the in-patients have to provide certain articles for themselves. These articles include the supply of tea, sugar, and butter, changes of personal linen, washing, &c. After full consideration they have, in increasing the grants or in renewing the larger grants made to institutions where these requirements are enforced, called the attention of the authorities to the objections attaching to the system, and have requested that the articles in question may in future be provided by the hospitals. Several of the best managed institutions make no such demands on the patients.

"The Committee's anticipation that the League of Mercy, instituted two years ago by H.R.H., would in due time greatly benefit the Fund, is confirmed by the fact that the Committee are enabled to advise the employment of £6,000 from that source, this being the amount which will be paid to the Treasurer by the League in connection with this year's distribution.

"In spite of the exceptionally large demands upon the charity of the public occasioned by the war, the contributions to the Fund have not shown any serious falling off during the past year; a gratifying proof of increasing recognition of the importance of the services rendered by the Fund. In consideration of the unusual needs of the several hospitals at the present time, it has been decided by the Executive Committee to place at our disposal for distribution to the hospitals this year £49,000, as compared with £41,000 distributed last year.

"It is recommended that the additional £8,000 now at the disposal of the Fund be employed mainly in the way of donations, because the special circumstances of the year have tended to lessen the amount given by the public to the support of individual hospitals, and it has been thought better to supplement these deficiencies by special donations for the current year, rather than to attempt to place additional beds at the disposal of the public, for which continuous maintenance would have to be provided. It must not be forgotten, however, that during the last three years 287 additional beds have been made available to the poor of the Metropolis by the agency of this fund."

### GRANTS RECOMMENDED BY THE HOSPITALS DISTRIBUTION COMMITTEE.

| Name of Hospital | Grants | | Remarks. |
|---|---|---|---|
| | Annual | Donation | |
| | £ | £ | |
| Alexandra Hospital for Children ... ... | — | 500 | |
| Belgrave Hospital ... ... ... ... | — | 500 | To help the Hospital to remove to the South of London. |
| Bolingbroke Hospital... ... ... ... | — | *600 | |
| British Lying-in Hospital ... ... ... | — | *200 | Laboratories to be renewed. |
| Cancer Hospital ... ... ... ... | — | *350 | |
| Central London Ophthalmic Hospital ... | — | — | Claim to be considered another year when Building commenced. |
| Central London Throat and Ear Hospital ... | — | — | Claim to be considered another year when Building commenced. |
| Charing Cross Hospital ... ... ... | — | 1,500 | Provided Wards are closed during reconstruction. |
| Chelsea Hospital for Women ... ... | — | *350 | £250 for reflooring Ward. |
| Cheyne Hospital ... ... ... ... | — | 100 | In consideration of the fact that curable cases are admitted. |
| City of London Hospital for Diseases of the Chest, Victoria Park, E. ... ... ... | 1,500 | *100 | To provide the maintenance for 25 beds. |
| City Orthopædic Hospital ... ... ... | 250 | *50 | The amount named by the authorities as required to maintain certain beds. |
| City of London Lying-in Hospital ... ... | — | 250 | For Nurses' dormitories. |
| Clapham Maternity Hospital ... ... | — | — | Well and economically managed. |
| Cottage Hospital, Canning Town ... ... | — | *125 | |
| Dental Hospital ... ... ... ... | — | 100 | |
| Dreadnought Hospital ... ... ... | 500 | 500 | £500 annual as before, £500 for the Tropical Diseases Department. |

GRANTS RECOMMENDED BY THE HOSPITALS DISTRIBUTION COMMITTEE—*continued.*

| | £ | £ | |
|---|---|---|---|
| East End Mothers' Home ... ... ... | — | 100 | For enlarging kitchen. |
| East London Hospital ... ... ... | — | 1,000 | £500 for new Mortuary and Dead House. |
| Establishment for Gentlewomen ... ... | — | 75 | |
| Evelina Hospital ... ... ... ... | — | — | No grant is recommended, as the income appears sufficient this year. |
| French Hospital ... ... ... ... | — | 100 | |
| German Hospital ... ... ... ... | — | 250 | |
| Gordon Fistula Hospital ... ... ... | — | 100 | Towards furnishing. |
| Great Northern Central Hospital ... ... | 750 | 500 | £250 towards new laundry. |
| Grosvenor Hospital for Women and Children ... ... ... ... ... | — | *150 | |
| Guy's Hospital... ... ... ... ... | †5,000 | — | |
| Hampstead ... ... ... ... ... | — | 250 | |
| Hospital for Diseases of the Throat ... | — | 100 | |
| Hospital for Epilepsy and Paralysis ... | — | †250 | For rebuilding. |
| Hospital for Sick Children ... ... ... | 500 | — | |
| Hospital for Women, Soho Square ... ... | — | *400 | |
| Hospital of St. John and St. Elizabeth ... | — | †250 | For Equipment Fund. |
| Italian ... ... ... ... ... ... | — | 100 | |
| King's College Hospital ... ... ... | 1,000 | *250 | |
| London Fever ... ... ... ... ... | — | 500 | Attention to be drawn to the necessity for certain reconstructions. |
| London Homœopathic Hospital ... ... | — | †200 | |
| London Hospital ... ... ... ... | †5,000 | — | |
| London Lock Hospital (Female) ... ... | — | †500 | Structural defects to be remedied. |
| London Temperance Hospital ... ... | — | 600 | Conditional upon appointment of additional surgeon. |
| London Throat ... ... ... ... | — | — | Unable to recommend a grant. |
| Memorial Cottage Hospital ... ... ... | — | *75 | |
| Metropolitan Hospital ... ... ... | — | 1,500 | The Hospital has been able to pay off the debt with the assistance and encouragement shown by last year's grant. |
| Middlesex Hospital ... ... ... ... | 1,000 | *250 | Hours of nurses' work too long. |
| Mildmay Mission Hospital ... ... ... | — | 250 | Mortuary to be provided. |
| Miller Hospital ... ... ... ... | — | *550 | |
| National Hospital for the Paralysed ... | 750 | *200 | Subject to settlement of the existing differences by May 1st, 1901. |
| National Orthopædic Hospital ... ... | — | — | No grant this year. |
| New Hospital for Women ... ... ... | — | *250 | |
| North-Eastern Hospital for Children ... | 500 | *550 | |
| North London Hospital for Consumption ... | 1,000 | *100 | |
| North-West London Hospital ... ... | — | 500 | Bath rooms a necessity. |
| Paddington Green Hospital for Children | — | 200 | |
| Poplar Hospital ... ... ... ... | — | *500 | For New Medical Wards, &c. |
| Passmore-Edwards' Hospital ... ... | — | *75 | |
| Queen Charlotte's Hospital ... ... ... | — | 300 | |
| Royal Ear Hospital ... ... ... ... | — | — | Unable to recommend a grant. |
| Royal Eye Hospital ... ... ... ... | — | *150 | |
| Royal Free Hospital ... ... ... ... | 750 | 750 | £500 a donation towards improving the accommodation for Nurses; £250 for new Lavatories. |
| Royal Hospital for Diseases of the Chest, City Road | — | *550 | £250 to Building Fund. |
| Royal Hospital for Women and Children ... | — | 500 | The Committee desire to draw attention to the need for rebuilding. |
| Royal London Ophthalmic Hospital ... | — | 1,250 | |
| Royal Orthopædic Hospital ... ... ... | — | — | No grant, last year's conditions being ignored. |
| Royal Westminster Ophthalmic ... ... | — | — | No grant this year. |
| Samaritan Free Hospital ... ... ... | 500 | *50 | |
| St. George's Hospital... ... ... ... | — | *1,000 | Special Donation. |
| St. Mark's Hospital ... ... ... ... | — | *150 | |
| St. Mary's Children's Hospital, Plaistow ... | — | 250 | For maintenance. |
| St. Mary's Hospital, Paddington .. ... | 1,000 | *200 | |
| St. Monica's Hospital... ... ... ... | — | 50 | An Isolation Ward has been provided. |
| St. Peter's Hospital for Stone ... ... | — | 50 | |
| St. Saviour's Hospital, Osnaburgh Street ... | — | 50 | |
| St. Thomas's Hospital ... ... ... | 1,800 | — | |
| Tottenham Hospital ... ... ... ... | 1,000 | — | Towards the maintenance of a ward containing about 15 beds. |
| University College Hospital... ... ... | 1,000 | *400 | In course of rebuilding. |
| Victoria Hospital for Sick Children ... | — | *150 | New building not yet commenced. |
| West End Hospital for Diseases of the Nervous System | — | *150 | Operating Room not yet provided. |
| Western Ophthalmic Hospital ... ... | — | — | Building unsuitable for hospital purposes. |
| West Ham Hospital ... ... ... ... | 300 | *600 | |
| West London Hospital ... ... ... | — | *1,150 | In hopes special efforts will be made to clear off debt. |
| Westminster Hospital ... ... ... | 750 | 500 | £750 annual; £500 towards the cost of out-patient Bar and new electrical equipment. |
| Wimbledon Hospital ... ... ... ... | — | — | A useful institution for local purposes. |

* These hospitals have been given special grants with the view to the abolition of the requirements imposed upon in-patients to supply certain articles of food, clothing, &c. † It is hoped that in these cases also a similar abolition will be made.

Mr. J. G. Craggs read the report of the Convalescent Homes Committee, which was as follows:—

In deciding upon the claims of the various convalescent homes, the Committee have to bear in mind that they have only a limited amount at their disposal, and that they are dealing with this sum under the terms of a special trust. In these circumstances it has been deemed advisable to follow the same course as last year by making the grants fairly substantial rather than by multiplying the number of participating institutions. This course necessitates the exclusion of many convalescent homes the names of which might under more favourable conditions have appeared in the following list. The Committee recommend the following grants:—

| | £ |
|---|---|
| Alexandra Hospital, Convalescent Home at Painswick | 50 |
| Chelsea Hospital for Women, Convalescent Home at St. Leonards-on-Sea | 50 |
| Charing Cross Hospital, Convalescent Home at Limpsfield, Surrey | 25 |
| East London Hospital for Children, Convalescent Home at Bognor | 100 |
| King's College Hospital, Convalescent Home at Hemel Hempstead | 100 |
| Mary Wardell Convalescent Home, Stanmore, Middlesex | 150 |
| Middlesex Hospital, Convalescent Home at Clacton-on-Sea | 100 |
| Metropolitan Convalescent Institution, 32 Sackville Street, Piccadilly, W. | 100 |
| Mrs. Kitto's Convalescent Home, Saith Park, Reigate | 50 |
| Metropolitan Hospital, Convalescent Home at Cranbrook | 50 |
| Morley House Seaside, Convalescent Home for Working Men at St. Margaret's Bay, Dover | 25 |
| National Hospital for the Paralysed and Epileptic, Convalescent Home at East Finchley | 50 |
| Paddington Green Children's Hospital, Convalescent Home at Harrow | 50 |
| Queen Charlotte's Lying-in Hospital, Convalescent Home at 20 Victoria Road, Kilburn | 50 |
| Victoria Hospital, Convalescent Home at Broadstairs | 50 |

For the Committe,

(Signed) William Latham.

December 12th, 1900.

H.R.H. The Prince of Wales: I have much pleasure in moving the adoption of the reports you have just heard read. I think we owe our deepest thanks to the gentlemen who have given so much of their valuable time to enquiring so fully into the needs of the metropolitan hospitals. We are distributing a much larger sum this year than we have ever done before, but I think we may be sure that it will be money well expended.

Sir William MacCormac seconded, and the reports were unanimously adopted.

A vote of thanks to the Prince of Wales for presiding was moved by the Lord Mayor, who said that he could assure His Royal Highness that the City still continued to take an unfailing interest in this important Fund. When His Royal Highness thought the fit and proper time had arrived for doing so, he would make another and perhaps a special appeal to the City for increased support and assistance.

Mr. J. Pierpoint Morgan, jun., seconded, and the vote was unanimously accorded.

His Royal Highness, in replying, said: I am glad to hear what the Lord Mayor has told us, and I am sure we all realise what a great thing it is for the Fund that the City of London should give us its hearty support. It gives me great pleasure to take this opportunity of thanking all those gentlemen who have assisted me in the great work of this institution—the members of the General Council, the visitors to the hospitals, the Executive Committee, and the honorary officers. I am very glad Lord Duncannon has been able to take Sir Savile Crossley's place, and I cannot say how much we are indebted to Mr. Craggs for the labours he gives gratuitously to the Fund. We have all plenty to do, so I will not keep you any longer; but I think we may separate to-day with the feeling that the Fund is in a very prosperous state, and that the interests of our great hospitals are still kept well before the public. I can only hope that as time goes on the Fund may increase in prosperity, so that the great object we had in view, that of placing the hospitals of this great Metropolis on a thoroughly sound financial basis, may be accomplished. (Applause.)

The meeting then terminated.

